# The 24 Hour Urine Creatinine Clearance for Prediction of Glomerular Filtration Rate in Liver Cirrhosis Patients: Have We Considered All Elements?

**DOI:** 10.5812/hepatmon.13398

**Published:** 2013-07-17

**Authors:** Mohammad Kazem Fallahzadeh, Neeraj Singh

**Affiliations:** 1 Division of Nephrology, Department of Medicine, LSUHSC-S, Shreveport, LA, USA; 2 WK John C. McDonald Regional Transplant Center, Shreveport, LA, USA

**Keywords:** Glomerular Filtration Rate, Liver Cirrhosis, Liver Transplantation, Kidney Function Tests

*Dear Editor,*

The correct estimation of renal function in patients with liver cirrhosis and chronic kidney disease (CKD) is difficult (1, 2). Direct measurement of glomerular filtration rate (GFR) using exogenous markers (iohexol or iothalamate) is considered the reference to assess renal function in cirrhotic patients (1, 2). However, direct measurement of GFR is not available at all transplant centers. The 24 hour urine creatinine clearance (CrCl) is widely used to measure GFR, and is calculated by multiplying the ratio of urine creatinine (Cr) to plasma Cr by 24 hours urine volume. However CrCl has several limitations. Besides the problem with accurately timed urine collection, CrCl is reported to overestimate the true GFR in liver cirrhosis patients compared with the direct measurement of GFR (1, 2). A low plasma Cr secondary to liver disease and poor muscle mass may overestimate GFR using CrCl. In addition, the GFR overestimation is also reported to be due to over-secretion of Cr from renal tubules in proportion to glomerular filtration, especially at low GFR (1, 2). Conversely, we have found that the total amount of Cr excreted in cirrhotic patients is lower than the minimum expected Cr excretion in 24 hours urine (20 mg/kg/day in males and 15 mg/kg/day in females). Hence, when urine Cr excretion is low, CrCl may also on the contrary underestimate the true GFR. In a retrospective study, we evaluated the charts of 160 consecutive patients who underwent liver transplantation alone (LTA) at our center from January 2002 to December 2012. Out of these, 25 patients had CKD with pre-transplant estimated GFR values ≤ 60 ml/min/ 1.73 m2 calculated using 4-variable and 6-variable modification of diet in renal disease (MDRD) equations. The 24-hour urine CrCl was available in all 25 patients within a month pre-transplant. Ten patients were excluded from analysis as their collected urine volumes were either < 750 ml or > 3000 ml suggesting under- or over- collection of urine. In remaining 15 patients, mean observed urine Cr excretion was significantly lower than the minimum expected Cr excretion in 24 hour urine (1.28 ± 0.62 grams/day vs. 1.69 ± 0.43 grams/day; P = 0.04). In these 15 patients with CKD, there was no significant difference between CrCl and MDRD-4 (49.6 ± 23.5 vs. 41.7 ± 11.6 respectively, P = 0.63), and between CrCl and MDRD-6 (49.6 ± 23.5 vs. 37.2 ± 9.5, respectively, P = 0.19) pre-transplant. However, GFR values at three months post-transplant were significantly higher compared with their corresponding values pre-transplant (see Table 1). The lower urine Cr excretion in these patients is probably secondary to decreased Cr production due to poor muscle mass and liver disease. The improvement in e-GFR early post-transplant suggests that there is likely some hemodynamic component to CKD pre-transplant. It is reported that the CKD eventually gets worse overtime post-LTA due to calcineurin inhibitor therapy and other risk factors (3, 4). In our study, although we did not measure GFR directly, the improved GFR values early post-LTA likely reflect true pre-transplant GFR values. In conclusion, although CrCl has been reported to overestimate GFR in liver cirrhosis patients with CKD, a lower than expected 24 hour urine creatinine excretion may also cause underestimation of GFR.

**Table 1.** Pre- and Post-Liver Transplant (LT) e-GFR Values in 25 Study Patients

e-GFR (ml/min/1.73m^2^)	Pre-LT	3 Months Post-LT	P Value
**MDRD-4**	41.9 ± 12.4	56.1 ± 20	0.016
**MDRD-6**	36.9 ± 10.4	52.3 ± 17.4	0.003
